# Molecular Mechanisms of Gonadal Differentiation Induced by 17β-Estradiol and Testosterone Propionate in *Rana dybowskii*

**DOI:** 10.3390/ani16152444

**Published:** 2026-08-06

**Authors:** Hualin Fu, Yanqiu Sun, Yiwen Sun, Xiangyu Meng, Wei Xu, Yuan Xu, Zhiheng Du

**Affiliations:** College of Animal Science and Technology, Northeast Agricultural University, No. 600 Changjiang Road, Xiangfang District, Harbin 150030, China; 15069496688@163.com (H.F.); s2751908995@163.com (Y.S.); 15135520115@163.com (Y.S.); mengxy0706@163.com (X.M.); 15937335908@163.com (W.X.)

**Keywords:** *Rana dybowskii*, 17β-estradiol, testosterone propionate, transcriptome, sex-biased genes, qRT-PCR

## Abstract

*Rana dybowskii* is an economically important amphibian in Northeast China, valued for its medicinal oviduct (*Oviductus Ranae*). However, the low proportion of females in artificial culture severely restricts industrial productivity. To elucidate the molecular mechanisms of hormone-induced sex reversal, based on our previous research results, we exposed tadpoles to 17β-estradiol (E_2_) or testosterone propionate (TP). We then monitored gonadal histology, conducted multi-stage transcriptome sequencing, and validated key genes via qRT-PCR. The results show that induction treatment with 40 μg·L^−1^ E_2_ and 80 μg·L^−1^ TP resulted in 93.33% female and 100% male populations, respectively. Both hormones promoted germ cell proliferation at the undifferentiated stage and directed stable ovarian or testicular development without intersex abnormalities. Ovarian differentiation involved progressive multi-wave transcriptional reprogramming, whereas testicular differentiation exhibited a concentrated gene expression burst during gonadal maturation. Steroid hormone biosynthesis, Wnt, and PPAR pathways formed the core regulatory network. Three female-biased genes (*3α-hsd*, *Adcy3*, *Rspo1*) and three male-biased genes (*Ptgs2*, *Sox9*, *Shbg*) were identified, with their expression dynamics aligned with histological progression. This study defines the optimal parameters and molecular signatures of bidirectional sex reversal in *R. dybowskii*. It provides a practical foundation for sex-control breeding and offers amphibian-based evidence for the conservation and plasticity of vertebrate sex determination.

## Simple Summary

The *Rana dybowskii* is widely farmed in Northeast China because its dried oviducts are used in traditional medicine. However, captive breeding usually produces a male-biased tadpole population, which severely restricts the yield and economic benefit of Oviductus Ranae. To address this issue, we attempted to treat tadpoles with sex hormones to control the population’s sex composition. However, the mechanisms by which sex hormones influence the sex determination of the Northeast Forest Frog remain poorly understood. Building upon previous research, we treated tadpoles with 40 μg·L^−1^ 17β-estradiol (E_2_) and 80 μg·L^−1^ testosterone propionate (TP), respectively, to investigate the molecular mechanisms underlying E_2_’s feminizing effect and TP’s masculinizing effect on the Northeast Forest Frog. Through transcriptome sequencing, we discovered that the hormones activated different sets of genes and biological pathways. Female development requires several waves of gene activity over time, whereas male development depended on a single, intense burst. We identified six key genes that drive this process. The findings provide a practical method to produce more female frogs, increasing profits and reducing wild collection, while also assisting scientists in understanding sex determination across animals.

## 1. Introduction

*Rana dybowskii* is an economically valuable amphibian species in Northeast China, distributed in Northeast China (Heilongjiang, Jilin, and northeastern Inner Mongolia), and also found in the Korean Peninsula and the Russian Far East. Its distinctive ecological habits and medicinal value have rendered it a key species in specialized aquaculture in Northern China. The dried oviduct of female *Rana dybowskii*, known as *Oviductus Ranae* (OR), is a highly regarded traditional Chinese medicine commonly used as a tonic. OR contains a variety of bioactive components with immunomodulatory, antioxidant, and anti-fatigue properties, which have supported its long-standing medicinal use and significant economic value [1]. However, under artificial culture conditions, the consistently low proportion of females has emerged as a crucial bottleneck restricting the profitability of the industry. The consistently male-biased sex ratio under artificial conditions may be attributed to several interacting factors. Elevated water temperatures during the temperature-sensitive period can suppress *Cyp19a1* expression and aromatase activity, thereby reducing endogenous estrogen synthesis and driving masculinization of genetic females [2,3,4,5]. High stocking densities—common in commercial farming—can activate stress-related endocrine pathways via the hypothalamic–pituitary–gonadal axis, perturbing the balance of endogenous sex steroids, while trace environmental endocrine-disrupting compounds in aquaculture water may further compromise normal gonadal differentiation. Although temperature can influence sex differentiation, its practical application is limited by inconsistent effects across studies, the technical difficulty and energy cost of precise temperature control in outdoor systems [2,6], and increased risks of malformation and mortality at extreme temperatures. In contrast, hormone-based sex control achieves highly predictable and efficient outcomes (over 93% females or 100% males), involves simple water-borne administration with regular renewal, incurs minimal operational costs, and offers dose-dependent controllability, making it the preferred strategy for commercial-scale monosex production in *R. dybowskii* [7,8]. Therefore, elucidating the sex-determination mechanisms and developing efficient sex-control technologies not only have direct practical implications for the sustainable development of *R. dybowskii* farming but also provide a unique perspective for exploring the evolution of sex-determination mechanisms in vertebrates.

Amphibians, occupying a pivotal phylogenetic position in vertebrate evolution, exhibit sex determination characterized by both genetic and environmentally sensitive features. Their gonadal differentiation is highly susceptible to modification by environmental factors such as temperature and exogenous hormones, making them an ideal model for investigating gene–environment interactions in sex determination. Vertebrate sex determination and differentiation are precisely regulated by both genetic and environmental cues [2,9,10,11,12]. In fish and amphibians, exogenous hormone treatments have been extensively shown to induce sex reversal within the “plastic window” of gonadal differentiation, thereby generating monosex populations. Studies have indicated that appropriate concentrations of 17β-estradiol (E_2_) or testosterone propionate (TP) can completely reverse individuals towards female or male phenotypes, with successful applications reported in *Oreochromis niloticus*, *Channa maculata*, anuran amphibians, and other species [7,13,14,15,16]. However, most studies focus on unidirectional sex reversal—especially estrogen-induced feminization—and a systematic, comparative molecular dissection of an efficient, complete bidirectional sex reversal system remains lacking. Most studies have only examined a limited number of well-known sex-determination candidate genes, such as *Dmrt1*, *Cyp19a1a* and *Foxl2* [17,18,19,20,21]. The optimal hormone concentrations for inducing complete sex reversal and the critical histological time points of gonadal fate transition remain undefined, which hinders the precise application of sex-control technologies and precludes a comprehensive understanding of the complex transcriptional reprogramming events and their regulatory networks during sex reversal [22,23,24,25].

The emergence of transcriptome sequencing technologies has provided powerful tools for systematically dissecting the molecular regulatory networks of sex determination and reversal. In recent years, by integrating histological observations with transcriptomic profiling, researchers have compared mature gonads between sexes or captured temporal transcriptomic dynamics during hormone-induced sex reversal in species such as *Channa maculata* [26], *Danio rerio* [27], and *Cyprinus carpio* [28]. These studies have identified core regulatory pathways, including TGF-β signaling, Wnt signaling, and steroid hormone biosynthesis, along with a set of sex-biased candidate genes [29,30,31,32]. Nevertheless, for *R. dybowskii*, the optimal hormone concentrations for inducing sex reversal, the histological dynamics of gonadal fate transition, and the underlying global transcriptional reprogramming events and key regulatory networks remain largely unknown. In particular, a comparative analysis of a “bidirectional sex reversal” system that achieves both efficient feminization and masculinization and its molecular basis has not been established in this species. Such bidirectional comparisons are essential for systematically revealing the common and specific molecular mechanisms underlying sex differentiation pathways.

Based on this background, the present study utilizes *R. dybowskii* tadpoles as the research model and combines histological and multi-stage transcriptomic analyses to systematically elucidate the molecular mechanisms of efficient bidirectional sex reversal induced by 17β-estradiol and testosterone propionate. The specific objectives were (1) to reveal the dynamic trajectory of gonadal transition towards ovaries or testes through time-course histological observation; (2) to construct a transcriptomic atlas across critical stages of gonadal differentiation and decipher the spatiotemporal expression characteristics of differentially expressed genes; and (3) to identify core signaling pathways and key candidate genes governing this process. This study not only provides essential theoretical foundations and precise technical parameters for sex-control breeding of *R. dybowskii* but also offers new scientific insights into the molecular networks by which environmental–genetic interactions regulate sex determination in amphibians and other vertebrates.

## 2. Materials and Methods

### 2.1. Animal Husbandry and Ethics Statement

Twenty batches of Northeast Forest Frog (*Rana dybowskii*) egg masses (spawned on the same day with identical developmental stages) were purchased from the Songfengshan Forest Frog Breeding Farm in Acheng District, Harbin City (45°32′ N, 126°58′ E). They were transported via a constant-temperature transport box (10–15 °C) to the Amphibian Breeding Laboratory at Northeast Agricultural University. After hatching, healthy embryos were selected for experimental use. Eleven treatment groups were established, with five replicates per group and 100 tadpoles per replicate. At the mouth-opening and opercular fold stages, a total of 5500 healthy tadpoles were randomly allocated into 55 white rectangular plastic containers (31.5 cm × 23.5 cm × 9.5 cm) filled with 3 L of aerated tap water (water depth approximately 4.5 cm). During the first 10 days post-hatching, tadpoles were fed colloidal egg masses every 12 h. Thereafter, they were fed granular tadpole feed once every 24 h at a fixed time.

Water quality parameters were monitored every 3 days throughout the experiment: pH = 7.2 ± 0.3, dissolved oxygen > 6.5 mg·L^−1^, total ammonia nitrogen < 0.02 mg·L^−1^, nitrite < 0.005 mg·L^−1^, and water temperature = 19 ± 1 °C. Laboratory environmental conditions were maintained at 19 ± 1 °C and 40 ± 5% relative humidity, and the photoperiod followed natural daylight cycles.

Note: The stocking density used in this study (~33 tadpoles·L^−1^) was higher than the 5–10 tadpoles·L^−1^ typically recommended for laboratory amphibian husbandry [33]. This density was intentionally chosen to approximate commercial *R. dybowskii* farming conditions in Northeast China, thereby enhancing the practical relevance of the results. While all experimental groups were maintained at identical densities to ensure internal validity, the potential interaction between high-density stress and hormone treatment warrants investigation in future studies.

### 2.2. Hormone Treatments and Sample Collection

17β-Estradiol (E_2_, 99%) and testosterone propionate (TP, 99%) were purchased from Shanghai Aladdin Biochemical Technology Co., Ltd. (Shanghai, China). Stock solutions of E_2_ and TP (99%, Aladdin, Shanghai, China) were prepared in absolute ethanol (final concentration ≤ 0.05% *v*/*v* in treatment groups). The control group (CK) received equivalent volumes of ethanol-diluted aerated tap water to control for solvent effects. The concentration gradient (10, 20, 40, 80, and 160 μg·L^−1^) was established based on a preliminary dose-range-finding experiment (0.5–500 μg·L^−1^), which identified 10–160 μg·L^−1^ as the safe and effective range for *R. dybowskii* tadpoles. The maximum concentration was substantially reduced compared with those employed in previous anuran immersion studies to avoid potential toxicity [15,16,34,35], and the five-point geometric progression (doubling steps) enabled robust dose–response curve fitting for optimal concentration determination. Accordingly, tadpoles were divided into 11 groups: a control group (CK), five E_2_ treatment groups (E_2_-10 to E_2_-160), and five TP treatment groups (TP-10 to TP-160), as detailed in Table 1 [36].

Hormones were administered via water-borne immersion continuously from the mouth-opening stage to metamorphic climax (stage 43). To determine the water renewal interval for maintaining hormone concentrations in this animal experiment, a quantitative analytical method for 17β-estradiol and testosterone propionate in water was established using an AB5500 QTrap (AB Sciex, Warrington, Cheshire, UK) triple quadrupole mass spectrometer. The results showed that the residual amounts of both hormones in the water exhibited a continuous decline over time, demonstrating significant time-dependent degradation characteristics. E_2_ retained >93% of its initial concentration after 24 h, while TP retained >66%; both declined considerably by 36 h. To ensure the consistency and stability of the experimental conditions, complete water changes were performed every 24 h in this study. The 24 h cycle therefore ensured that hormone levels remained above 50% of the nominal values throughout the experiment.

The tadpoles were reared until metamorphic climax (stage 43). Subsequently, 50 individuals per replicate per group were randomly sampled and fixed in Bouin’s fixative for anatomical observation of gonadal phenotype to ascertain gonadal sex and calculate the sex proportion.

During the tadpole rearing period, when the tadpoles in each treatment group reached stages 28, 30, 31, 35, 37, 40, and 43, three individuals were randomly selected from each group, placed into enzyme-free cryovials, rapidly frozen in liquid nitrogen, and then transferred to a −80 °C freezer for storage. Based on the sex identification results at stage 43, we selected the samples from stages 28 to 43 of the control group, the treatment group with the highest female ratio (E_2_-40), and the treatment group with the highest male ratio (TP-80) for transcriptome sequencing. Additionally, five tadpoles per treatment per stage were fixed in Bouin’s fixative for paraffin sectioning to observe morphological alterations during gonadal differentiation. Sex identification at Gosner stage 43 was performed based on gross gonadal morphology under a stereomicroscope (Leica M205C, Leica Microsystems, Wetzlar, Germany). Ovaries were identified by their elongated, lobulated morphology with a granular cortical surface and a visible cortical region enveloping the medulla; testes were identified by their shorter, smooth, cylindrical morphology with a translucent medullary region and the absence of cortical thickening.

### 2.3. Gonadal Histology

Gonadal tissues with the adjacent mesonephros were dissected under a stereomicroscope and fixed in Bouin’s fixative for 24 h at room temperature. Following fixation, tissues were dehydrated through a graded ethanol series (70%, 80%, 90%, 95%, and 100% ethanol) using an automated tissue processor (JJ-12J, Wuhan Junjie Electronics Co., Ltd., Wuhan, China), cleared in xylene, and embedded in paraffin. Serial transverse sections were cut at a thickness of 4 μm using a rotary microtome (RM2016, Leica Instruments, Shanghai, China). Sections were stained with hematoxylin and eosin (H&E), dehydrated through graded ethanols, cleared in xylene, and mounted with neutral balsam. Stained sections were examined and photographed under a light microscope (Axio Imager 2, Carl Zeiss, Oberkochen, Germany) equipped with a digital camera at magnifications of 100× and 200×. For each individual (n = 5 per treatment group per developmental stage), at least three non-consecutive sections spanning the mid-gonadal region were analyzed. Key histological features—including germ cell distribution, corticomedullary zonation, oocyte developmental stage, ovarian cavity formation, and seminiferous tubule organization—were assessed qualitatively. Gonadal sex was determined based on established histological criteria: ovaries were identified by the presence of a thickened cortex containing diplotene oocytes and an ovarian cavity; testes were identified by the presence of medullary seminiferous tubules containing spermatogonia and the absence of a prominent cortex.

### 2.4. RNA Sequencing and Transcriptomic Analysis

Total RNA was extracted from whole *R. dybowskii* tadpoles using TRIzol^®^ Reagent (Invitrogen, Carlsbad, CA, USA) in accordance with the manufacturer’s instructions. The concentration and purity of RNA were determined using a NanoDrop 2000 spectrophotometer (Thermo Fisher Scientific, Waltham, MA, USA), and the integrity of RNA was evaluated using an Agilent 5400 (Agilent Technologies, Santa Clara, CA, USA) Fragment Analyzer (Agilent Technologies, Santa Clara, CA, USA). Only high-quality total RNA samples (OD260/OD280 = 1.8–2.2, RNA integrity number (RIN) ≥ 8.0, total RNA ≥ 1 μg) were employed for subsequent library construction.

Transcriptome libraries for each sample were prepared using the Illumina^®^ Stranded mRNA Prep, Ligation kit (Illumina, San Diego, CA, USA). In brief, mRNA was isolated from total RNA using Oligo (dT)-coupled magnetic beads and fragmented. Utilizing the fragmented mRNA as a template, first-strand cDNA was synthesized with random hexamer primers and reverse transcriptase, followed by the synthesis of second-strand cDNA. The resultant double-stranded cDNA was subjected to end-repair, A-tailing, and adapter ligation. Adapter-ligated cDNA fragments were purified, size-selected, and enriched by PCR to construct strand-specific cDNA libraries. The fragment size distribution was assessed using a Bioanalyzer (Agilent Technologies, Santa Clara, CA, USA), and the effective library concentration was quantified by Q-PCR. Qualified libraries were sequenced on the NovaSeq X Plus platform (Illumina, San Diego, CA, USA) using a paired-end 150 bp (PE150) strategy. Library construction and sequencing were conducted by Shanghai Majorbio Bio-pharm Technology Co., Ltd., Shanghai, China.

### 2.5. Differential Expression Analysis and Functional Enrichment

To comprehensively characterize the gene expression dynamics during the gonadal differentiation and development of *R. dybowskii* tadpoles, a systematic differential expression analysis was carried out on the transcriptome data from the E_2_-40 group, the TP-80 group, and the control group across seven key developmental stages (28, 30, 31, 35, 37, 40, and 43). Gene expression levels were quantified using RSEM software (v1.3.3) [37] and normalized as fragments per kilobase of transcript per million mapped reads (FPKM). Principal component analysis (PCA) and Pearson correlation analysis were conducted via R base packages to evaluate the correlation of biological replicates within groups and the overall expression differences between groups.

Differentially expressed genes (DEGs) were identified through the DESeq2 R package (version 1.26.0) [38]. DESeq2 employs a negative binomial generalized linear model to assess the significance of gene expression differences between comparison groups, with *p*-values adjusted for multiple testing using the Benjamini–Hochberg method to control the false discovery rate (FDR). Genes with |log_2_ fold change| ≥ 1 and FDR < 0.05 were defined as significantly differentially expressed. KEGG pathway enrichment analysis was performed to clarify the functions and signaling pathways involving DEGs; KEGG analysis identified pathways related to signal transduction and metabolism.

### 2.6. Quantitative Real-Time Polymerase Chain Reaction (qRT-PCR) Validation

To validate the reliability of the transcriptome data and ascertain the expression patterns of candidate sex-biased genes, quantitative real-time PCR (qRT-PCR) was utilized to detect the expression levels of six selected differentially expressed genes (DEGs) (*3α-hsd*, *Adcy3*, *Rspo1*, *Ptgs2, Sox9*, and *Shbg*) at key developmental stages (31, 35, 37, and 40). *GAPDH* was employed as the internal reference gene. Primer sequences (Table 2) were synthesized by Sangon Biotech (Shanghai) Co., Ltd., Shanghai, China. Total RNA (1 μg) from the same batch of samples used for transcriptome sequencing was reverse-transcribed into cDNA using the HiScript III RT SuperMix for qPCR (+gDNA wiper) kit (Vazyme Biotech, Nanjing, China).

qRT-PCR reactions were conducted on a QuantStudio 6 Flex Real-Time PCR System (Applied Biosystems, USA) using ChamQ Universal SYBR qPCR Master Mix (Vazyme Biotech, Nanjing, China). The total reaction volume was 15 μL, comprising 7.5 μL of 2 × SYBR Green Master Mix, 0.4 μL of each forward and reverse primer (10 μM), an appropriate quantity of cDNA template, and sterile ultrapure water. The thermal cycling protocol was as follows: 95 °C for 2 min; followed by 40 cycles of 95 °C for 10 s and 60 °C for 30 s.

Relative gene expression was calculated using the 2^−ΔΔCt^ method [39], normalized to the Ct values of *GAPDH*, with *Adcy3* expression in the TP-80 group at stage 37 serving as the calibrator. Differences in target gene expression between the E_2_-40 and TP-80 groups at the same developmental stages were compared to verify sex-biased expression. Expression patterns were examined, and the acquired data were analyzed for consistency with the transcriptome sequencing results to guarantee the accuracy and reliability of the sequencing data.

### 2.7. Data Analysis

All data are presented as the means ± standard deviation (SD). One-way analysis of variance (ANOVA) was carried out using IBM SPSS Statistics 26.0 (IBM, Armonk, NY, USA) software, with *p* < 0.05 regarded as statistically significant. Graphs were generated via GraphPad Prism 9.0 (GraphPad Software, San Diego, CA, USA), and transcriptomic analyses were performed using R 4.2.1.

## 3. Results

### 3.1. Effects of Hormone Treatment on the Sex Proportion of R. dybowskii

**Figure 1:**
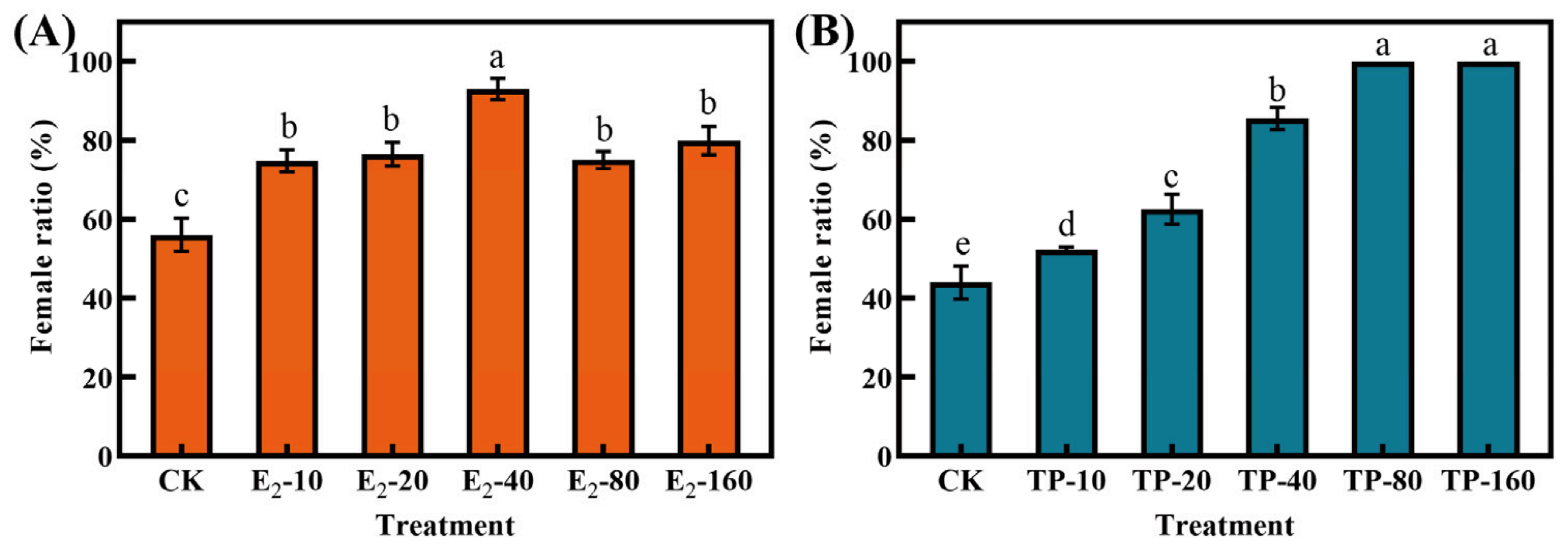
Effect of exogenous hormone treatment on the sex ratio of *R. dybowskii*: (**A**) Proportion of females in 17β-estradiol treatment groups. (**B**) Proportion of males in testosterone propionate treatment groups. Data are presented as means ± standard deviation (n = 5 replicates, 50 individuals per replicate). Note: Different lowercase letters denote significant differences among hormone levels (*p* < 0.05).

The effects of 17β-estradiol and testosterone propionate on the sex proportion of *R. dybowskii* are presented in Figure 1. 17β-estradiol treatment significantly induced female differentiation (Figure 1A). As the E_2_ concentration increased from 10 μg·L^−1^ to 40 μg·L^−1^, the female proportion progressively rose, reaching a maximum of 93.33% in the E_2_-40 group—a 37.50 percentage-point increase compared with the control group (55.83% vs. 93.33%; this corresponds to a 67.17% relative increase over the control value). When the concentration was further increased to 80 μg·L^−1^ and 160 μg·L^−1^, the female proportion declined, indicating that 40 μg·L^−1^ is the optimal concentration for feminization. When the concentration was further increased to 80 μg·L^−1^ and 160 μg·L^−1^, the female proportion declined compared with the 40 μg·L^−1^ treatment, indicating a biphasic dose–response. However, the female proportion at these higher concentrations (80 μg·L^−1^: 75.00%; 160 μg·L^−1^: 79.93%) remained significantly above the control level (55.83%), demonstrating that the feminizing effect, while attenuated, was not reversed. Conversely, testosterone propionate effectively directed gonadal differentiation of tadpoles toward the male phenotype (Figure 1B). With increasing TP concentration, the male proportion of *R. dybowskii* gradually increased. The TP-10 group showed an 8.33% increase in male ratio compared to the control; both the TP-80 and TP-160 groups achieved a 100% male ratio. Therefore, 80 μg·L^−1^ TP represents the minimum effective concentration for complete masculinization. Based on the principle of minimizing chemical input in aquaculture applications, 80 μg·L^−1^ is recommended as the optimal concentration for production use. Subsequent studies selected the CK, E_2_-40, and TP-80 treatment groups, which exhibited the most pronounced differences in sex ratio.

### 3.2. Histological Analysis of Gonadal Development Under Hormone Treatment

Histological examination of *R. dybowskii* gonads from stages 28 through 43 revealed the developmental trajectory of hormone-induced sex reversal. Gonadal development proceeded through three phases: undifferentiated (stages 28–31), differentiation (stages 35–37), and maturation (stages 40–43).

**Figure 2:**
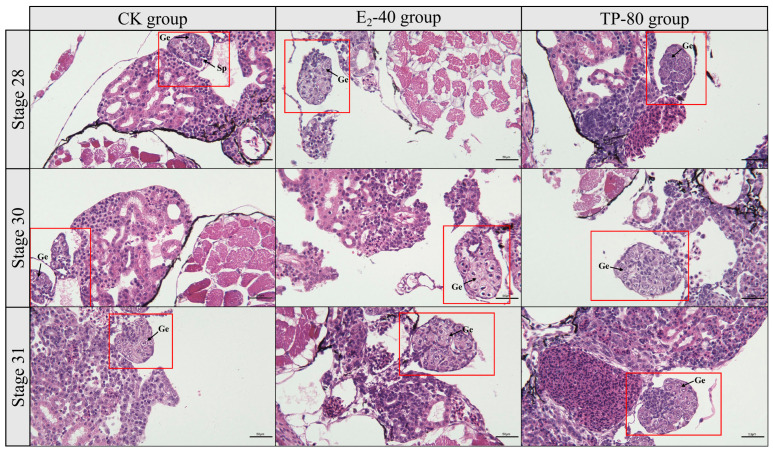
Histological analysis of gonadal development during the undifferentiated phase (stages 28–31) under hormone treatment, Red boxes mark the gonadal primordium tissue for histological observation; Ge = gonad, Sp = spermatogenic tissue.

During the undifferentiated phase (stages 28–31) (Figure 2), the gonadal primordium appeared as a paired ridge on the ventrolateral mesonephros in all groups. At stage 28, primordial germ cells (PGCs), identifiable by their large, spherical morphology and prominent nucleoli, were more abundant in the E_2_-40 and TP-80 groups than in the control, indicating early hormone-induced germ cell proliferation. Initial corticomedullary zonation emerged at stage 30 and became unequivocally established by stage 31, when germ cells were clustered predominantly within the cortex. In the E_2_-40 group, the cortex was thickened with densely packed oogonia, whereas in the TP-80 group, a subset of germ cells associated with developing medullary cords, foreshadowing testicular commitment.

**Figure 3:**
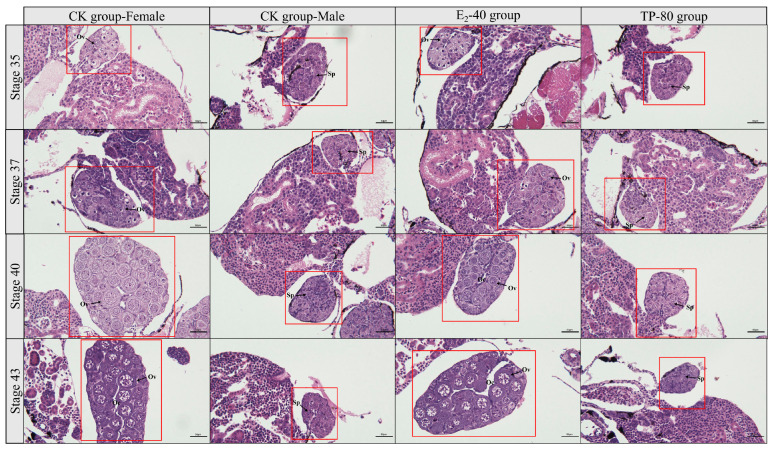
Histological analysis of gonadal differentiation and maturation (stages 35–43) under hormone treatment, Red boxes enclose the dissected gonadal tissue for morphological assessment; Ov = ovary, Sp = spermatogenic tissue, Oc = ovarian cavity.

In Stage 35 (Figure 3)—the stage at which clear histological dimorphism was first observed in the control group—among the E_2_-40 group, 93.25% of individuals developed ovarian morphology characterized by cortical thickening containing diplotene oocytes and well-defined ovarian lumen boundaries, whereas approximately 6.75% of individuals exhibited testicular morphology, featuring developing medullary seminiferous tubules and spermatogonia, consistent with the male phenotype observed in the control group; conversely, all TP-80 individuals displayed testicular morphology, manifested as reduced cortical thickness, expanded medulla, and well-defined spermatogenic tubules composed of support cells and spermatogonia; in none of the treatment groups was either intersexual or ambiguous gonadal phenotype observed.

During the maturation phase (stages 40–43) (Figure 3), ovarian and testicular differentiation intensified progressively. In the E_2_-40 group, the ovarian cavity expanded, oocytes increased in diameter, and follicular organization matured, while in the TP-80 group, seminiferous tubules underwent structural refinement with organized spermatogenic cysts. By stage 43 (metamorphic climax), the gonadal histology of E_2_-40 and TP-80 individuals was indistinguishable from that of control females and males, respectively. These observations demonstrate that 40 μg·L^−1^ E_2_ and 80 μg·L^−1^ TP induced complete, histologically normal phenotypic sex reversal without detectable gonadal abnormalities.

### 3.3. Transcriptome Landscape of Gonads During Hormone-Induced Sex Differentiation

**Figure 4:**
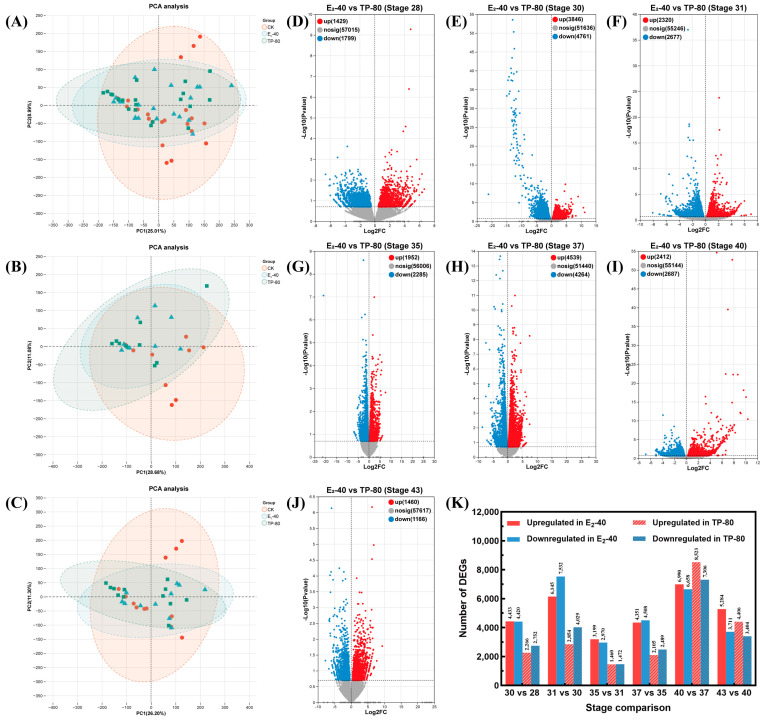
Transcriptomic characteristics of gonads during hormone-induced sex reversal. (**A**) Principal component analysis (PCA) of all samples across all stages; (**B**) PCA of samples at the undifferentiated stage (28–31); (**C**) PCA of samples at the differentiation stage (35–43); (**D**–**J**) volcano plots of DEGs between the E_2_-40 and TP-80 groups at stages 28, 30, 31, 35, 37, 40, and 43; (**K**) quantities of DEGs between adjacent stages within each treatment group.

To explore the molecular regulatory mechanisms underlying hormone-induced sex differentiation in *R. dybowskii* tadpoles, transcriptome sequencing was conducted on gonadal tissues from the control, E_2_-40, and TP-80 groups across seven crucial developmental stages (28–43), with three biological replicates per group per stage. A total of 63 samples were analyzed, generating 412.36 Gb of clean data, with each sample having more than 5.89 Gb. The Q30 percentage was above 95.97%. Clean reads from each sample were mapped to the *R. dybowskii* reference genome, with mapping rates exceeding 97% (Table 3), suggesting that the sequencing data were of reliable quality and appropriate for downstream analysis. Principal component analysis (PCA) unveiled the transcriptomic remodeling characteristics induced by hormones. The whole-period analysis (Figure 4A) indicated that PC1 and PC2 accounted for 25.01% and 8.99% of the total variance, respectively. The TP-80 group clustered independently on the right side along the PC1 axis, while the E_2_-40 and control groups overlapped. This suggests that TP induced a distinct masculinizing transcriptional program, whereas the E_2_-induced transcriptome closely resembled the natural differentiation state.

The analysis concentrating on the undifferentiated stage (stages 28–31) (Figure 4B) demonstrated even more prominent inter-group separation, with the explanatory rate of PC1 increasing to 28.68%. This indicates that hormones had commenced reshaping the transcriptional trajectory prior to the initiation of morphological differentiation.

In the analysis focusing on the differentiation and maturation stages (35–43) (Figure 4C), samples from the same developmental stages clustered more closely. Differential expression analysis identified a substantial number of differentially expressed genes (DEGs) across various comparison groups.

Across all seven stages (E_2_-40 vs. TP-80), a total of 37,597 DEGs were identified. Among these, the number of DEGs reached a peak at stage 30 (8607) and stage 37 (8803), which was a three- to four-fold increase compared to stage 28 (2145). This suggests that these two periods are crucial transcriptional nodes for sex differentiation.

Time-course comparisons (Figure 4K) showed that under the E_2_-40 treatment, the number of DEGs peaked at stage 31 vs. 30 (13,677) and stage 40 vs. 37 (13,648). Under the TP-80 treatment, a significant surge in differentially expressed genes (DEGs) was detected at stage 40 vs. 37 (15,829), with upregulated genes being predominant. These findings suggest that stages 30–31 (the transition from the undifferentiated state to differentiation) and stages 37–40 (gonadal structural maturation) are crucial windows for gene expression regulation. Time-course analysis demonstrated distinct regulatory rhythms between the male and female differentiation pathways. The E_2_-40 group displayed two transcriptional reprogramming peaks, whereas the TP-80 group exhibited a single burst. This pattern of gradual, multi-wave reprogramming (ovary) versus concentrated burst activation (testis) fundamentally reflects the distinct molecular dynamics underlying the sex differentiation pathways. In conclusion, ovarian development entails sustained, multi-wave transcriptional reprogramming, while testicular development experiences a concentrated transcriptional burst during the critical period of structural remodeling.

### 3.4. Signaling Pathway Enrichment Analysis in Sex-Differentiated Gonads

**Figure 5:**
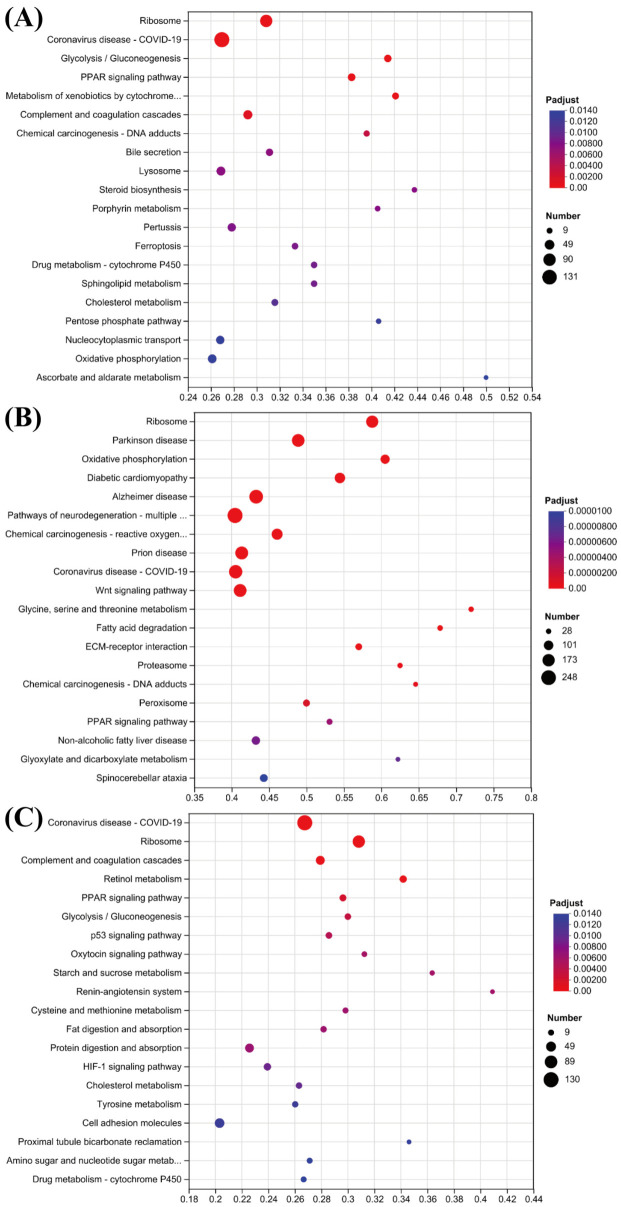
KEGG pathway enrichment analysis of differentially expressed genes. (**A**) Comparison between E_2_-40 and TP-80 at stage 31; (**B**) comparison between E_2_-40 and TP-80 at stage 37; (**C**) comparison between E_2_-40 and TP-80 at stage 40. The size of the bubble represents the number of enriched genes; the color represents the enrichment significance (adjusted *p*-value).

**Figure 6:**
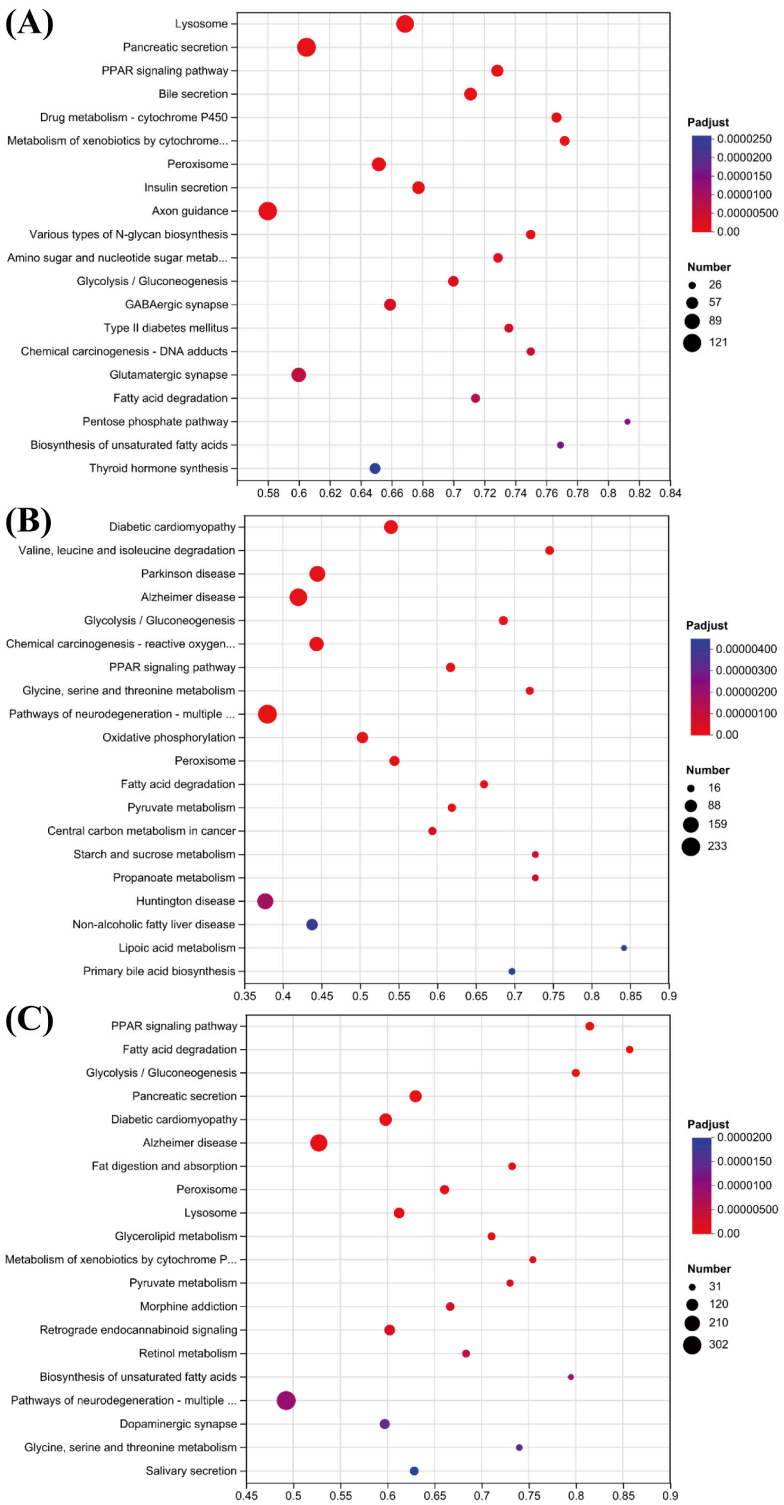
KEGG pathway enrichment analysis of differentially expressed genes. (**A**) In the E_2_-40 group: Comparison between stage 31 and stage 30. (**B**) In the E_2_-40 group: Comparison between stage 37 and stage 35. (**C**) In the E_2_-40 group: Comparison between stage 40 and stage 37. The size of the bubble represents the number of enriched genes; the color represents the enrichment significance (adjusted *p*-value).

**Figure 7:**
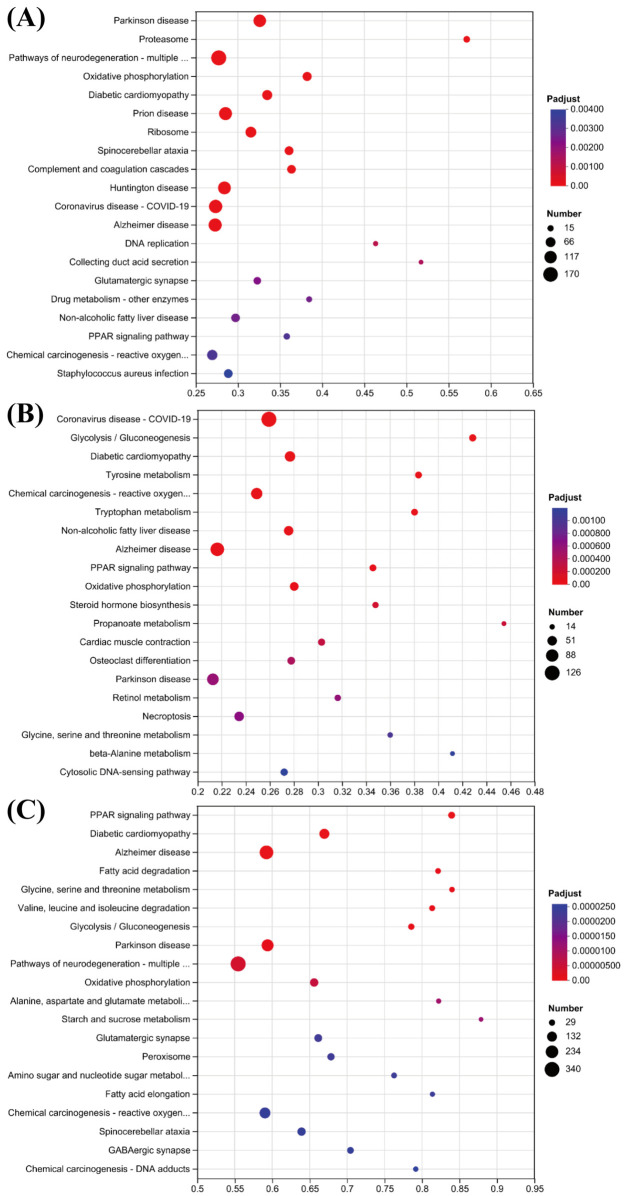
KEGG pathway enrichment analysis of differentially expressed genes. (**A**) In the TP-80 group: Comparison between stage 31 and stage 30. (**B**) In the TP-80 group: Comparison between stage 37 and stage 35. (**C**) In the TP-80 group: Comparison between stage 40 and stage 37. The size of the bubble represents the number of enriched genes; the color represents the enrichment significance (adjusted *p*-value).

Based on the dynamic expression patterns of the differentially expressed genes (DEGs), Kyoto Encyclopedia of Genes and Genomes (KEGG) pathway enrichment analysis was further conducted to identify the core molecular pathways governing sex reversal. KEGG enrichment analysis unveiled the core regulatory networks involved in sex reversal. Same-stage pathway enrichment analysis between the E_2_-40 and TP-80 groups (Figure 5) indicated that steroid hormone biosynthesis (KEGG ID: ko00140), Wnt signaling pathway (ko04310), and Peroxisome Proliferator-Activated Receptor (PPAR) signaling pathway (ko03320) were significantly enriched at both developmental stages 31 and 37 (adjusted *p* < 0.001), which constituted the core pathways for sex differentiation regulation. Among these, steroid hormone biosynthesis had the highest number of enriched DEGs at stage 31 (42 genes), including key genes such as *CYP19a1* and 3α-hydroxysteroid dehydrogenase (*3α-hsd*), which directly respond to exogenous hormone stimulation. Time-course enrichment analysis revealed significant differences in pathway regulation patterns between male and female differentiation. During estrogen-induced development, the Extracellular Matrix (ECM)–receptor interaction (ko04512) and Phosphatidylinositol 3-Kinase (PI3K)–Protein Kinase B (Akt) signaling pathway (ko04151) were significantly enriched at stage 35 compared to stage 31 (differentiation initiation) and stage 43 compared to stage 40 (maturation), participating respectively in gonadal structural construction and oocyte maturation. The PPAR signaling pathway was continuously enriched from stages 31 to 40, where it regulated lipid metabolism to provide energy for ovarian development (Figure 6). During TP-induced development, the steroid hormone biosynthesis and PPAR signaling pathways exhibited the highest enrichment at stages 37–40, offering the hormonal microenvironment and metabolic support for testicular tubular structure formation, which is highly congruent with the transcriptomic characteristics of the burst of DEGs during this period (Figure 7). Collectively, these findings suggest that the steroid hormone biosynthesis, Wnt, and PPAR signaling pathways form the core regulatory network of sex reversal, whereas the differential enrichment of the PI3K-Akt and ovary/testis-specific pathways reflects a fundamental division of labor in molecular regulation between male and female differentiation pathways, presenting distinct enrichment patterns according to the sex differentiation direction.

### 3.5. Screening and Identification of Sex-Biased Candidate Genes

Based on gene expression analysis and KEGG pathway enrichment outcomes, and taking into account gene expression patterns during gonadal development as well as their known functions, candidate genes were screened according to the following criteria: (1) significantly differentially expressed between the E_2_-40 and TP-80 groups at most critical developmental stages (|log_2_FC| ≥ 1, FDR < 0.05); (2) expression pattern consistent with the histological progression of gonadal differentiation; (3) enriched in KEGG core pathways (steroid hormone biosynthesis, Wnt, PPAR); and (4) known functions associated with sex differentiation. Six candidate genes closely related to sex determination and gonadal differentiation in *R. dybowskii* tadpoles were identified, including three genes with female-biased expression (*3α-hsd*, *Adcy3*, *Rspo1*) and three genes with male-biased expression (*Ptgs2*, *Sox9*, *Shbg*).

These genes demonstrated significant sexually dimorphic expression patterns across four crucial developmental stages, namely pre-differentiation, differentiation initiation, differentiation reinforcement, and structural maturation (stages 31, 35, 37, and 40). The three genes with female-biased expression showed notably higher expression levels in the E_2_-40 group compared to the TP-80 group; in particular, *Rspo1* was significantly upregulated from stage 31 onwards (log_2_FC = 2.14, FDR < 0.001), serving as an early marker of female differentiation. The three genes with male-biased expression exhibited significantly higher expression levels in the TP-80 group than in the E_2_-40 group; *Sox9* expression continuously increased. Subsequent to stage 35, it was fully synchronized with the process of testicular structural development. Functional annotation (Table 4) demonstrated that *3α-hsd* is involved in steroid hormone metabolism, catalyzing the conversion of estrogen precursors; *Adcy3* regulates oocyte maturation through the cAMP signaling pathway; *Rspo1* functions as an activator of the Wnt pathway, facilitating ovarian differentiation; *Ptgs2* serves as a crucial rate-limiting enzyme in prostaglandin synthesis, regulating spermatocyte proliferation; *Sox9* is a core transcription factor for testicular development; and *Shbg* maintains the homeostasis of free testosterone. These six genes were enriched in core pathways such as steroid hormone biosynthesis, Wnt, and PPAR, establishing a complete regulatory chain of “hormone signal–pathway regulation–structural establishment.”

### 3.6. qRT-PCR Validation of Candidate Gene Expression Patterns

**Figure 8:**
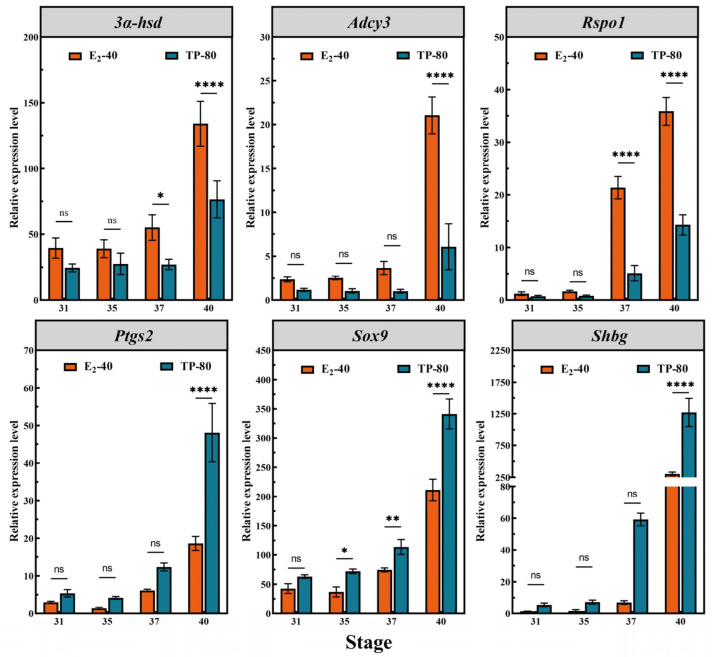
Relative expression levels of sex-biased genes validated by qRT-PCR. Relative expression was calculated using the 2^−ΔΔCt^ method with *GAPDH* as the internal reference. Data are presented as means ± SD (n = 3). ns: *p* ≥ 0.05, *: *p* < 0.05, **: *p* < 0.01, ****: *p* < 0.0001, indicating significant differences between the E_2_-40 and TP-80 groups at the same stage. ns.

The qRT-PCR results (Figure 8) indicated that the expression trends of all candidate genes were in accordance with the transcriptome sequencing data. The female-biased genes *3α-hsd*, *Adcy3*, and *Rspo1* were significantly more highly expressed in the E_2_-40 group across stages 31, 35, 37, and 40, with the exception that *3α-hsd* at stage 35 and *Rspo1* at stage 31 exhibited no significant differences. The male-biased genes *Ptgs2*, *Shbg*, and *Sox9* were all significantly more highly expressed in the TP-80 group across all four stages (*p* < 0.01), demonstrating distinct male-specific expression characteristics.

## 4. Discussion

### 4.1. Dose–Response Characteristics of Estrogen and Androgen Treatments

The success of hormone-induced sex reversal is critically dependent on precise dosage control [7,15]. In the present study, exposure to 40 μg·L^−1^ of E_2_ resulted in a 93.33% female proportion in *R. dybowskii*, while a treatment of 80 μg·L^−1^ of TP led to complete masculinization. This dose–response relationship reveals a biphasic characteristic specific to estrogen action in *R. dybowskii*: the feminizing effect increased to a maximum at 40 μg·L^−1^ E_2_ and then attenuated at higher concentrations. In contrast, the masculinizing effect of TP exhibited a monotonic dose–response within the tested range—complete masculinization was achieved at 80 μg·L^−1^ and maintained at 160 μg·L^−1^ without apparent attenuation, indicating that the androgen and estrogen signaling pathways are subject to distinct regulatory constraints in this species. The 80 μg·L^−1^ TP concentration therefore represents the minimum effective dose for complete masculinization. This asymmetry between feminizing and masculinizing dose–response profiles has been noted in other anuran sex-reversal studies and may reflect fundamental differences in receptor saturation kinetics, metabolic clearance rates, or negative feedback sensitivity between the two hormonal axes.

Analogous phenomena have been documented in *Channa maculata*; a dosage of 30 mg/kg of E_2_ induced a 70% sex reversal, whereas 60 mg/kg, despite accelerating early differentiation, caused gonadal regression in certain individuals [14]; in the *Chinese longsnout catfish*, a treatment of 10 mg/kg of E_2_ from 25 to 75 days post-hatching (dph) resulted in 100% feminization, yet higher concentrations significantly inhibited growth [14,32]. This dose-dependent effect is presumably associated with the saturation kinetics of hormone receptors: when exogenous hormone concentrations surpass the upper limit of receptor binding capacity, surplus hormone molecules may disrupt other signaling pathways through non-specific binding or inhibit endogenous hormone synthesis via negative feedback [40,41]. Kamińska et al. (2024) demonstrated that androgens collaborate with the Notch signaling pathway in the seminiferous epithelium to modulate genes associated with germ cell development and apoptosis [41]; overly high androgen concentrations have the potential to disrupt this equilibrium, thereby triggering apoptosis. Notably, within the concentration range tested in the present study, TP maintained a stable masculinizing effect. Full phenotypic masculinization was achieved at 80 μg·L^−1^ and persisted at 160 μg·L^−1^ with no significant reduction in efficacy. From the perspective of breeding applications, the concentrations of 40 μg·L^−1^ E_2_ and 80 μg·L^−1^ TP determined in this study achieved complete phenotypic sex reversal at the gonadal level by Gosner stage 43, as confirmed by both gross morphological and histological examination. No significant growth inhibition was detected in these treatment groups relative to the controls (See Supplementary Tables S1 and S2). However, the long-term stability of the reversed gonadal phenotype—particularly through sexual maturation and into reproductive adulthood—remains to be evaluated. Studies tracking hormone-treated individuals to reproductive age, as well as experiments examining gonadal morphology following hormone withdrawal, will be necessary to confirm the permanence of sex reversal and to fully validate the applicability of these parameters for aquaculture practice. In contrast to previous studies that solely concentrated on unidirectional sex induction [42,43], this study concurrently establishes precise parameters for bidirectional sex reversal, offering a generalizable technical framework for constructing monosex populations in amphibians.

### 4.2. Hormonal Effects During the Pre-Differentiation Stage Reshape the Sex-Determination Timeline

Histological observations indicated that during the undifferentiated stage (stages 28–31), the density of germ cells in the hormone-treated groups was already significantly higher than that in the control groups. This suggests that exogenous hormones initiated cellular regulation prior to the traditionally defined onset of gonadal differentiation. This finding advances the effective window of hormone action to the pre-differentiation stage, implying that the commitment to gonadal differentiation occurs earlier than the histologically detectable morphological differentiation. This is highly consistent with the results in *Channa maculata*, where E_2_ treatment promoted germ cell proliferation as early as 20 days post-fertilization [26]. From the perspective of developmental biology, this phenomenon suggests that exogenous hormones do not merely “switch” the sex program at the differentiation node; instead, they augment the germ cell pool at an earlier stage, furnishing more abundant cellular reserves for subsequent gonadal development. Nevertheless, the causal relationship between this enhanced proliferation and directed differentiation necessitates further verification via cell lineage tracing or proliferation inhibition experiments [44,45]. Functional studies have demonstrated that the androgen receptor (AR) affects germ cell migration and apoptosis by regulating PRMT6 expression, providing molecular insights into how hormones govern germ cell fate. This “reserve first, direct later” strategy might be a prerequisite for hormones to achieve complete and stable sex reversal. It should be noted, however, that our assessment of increased germ cell density was based on qualitative histological observations. Direct quantification of mitotic indices is required to confirm enhanced proliferation. Demonstrating that both E_2_ and TP treatments increased germ cell number in the developing gonads of five anuran species (*Rana temporaria, Bufo bufo, Xenopus laevis, Pelophylax esculentus,* and *Pelophylax lessonae*) and providing direct amphibian evidence for hormone-induced germ cell proliferation prior to morphological differentiation [7], Saidapur reported that TP treatment in *Rana curtipes* induced early germ cell proliferation in the medullary region, consistent with the onset of testicular differentiation [15]. These findings lend support to our interpretation, but the underlying cellular mechanisms in *R. dybowskii* remain to be elucidated.

From these observations, it is unclear whether the enhanced germ cell proliferation induced by exogenous hormones could lead to gonadal morphological abnormalities, given that early embryonic proliferative activity is typically under strict genetic control. In the present study, despite the qualitatively higher germ cell density observed in the E_2_-40 and TP-80 groups during the undifferentiated phase (stages 28–31), no gonadal malformations, intersex phenotypes, ovotestis formation, or gonadal dysgenesis were detected by serial histological examination across any subsequent developmental stage (stages 35–43; Figure 2 and Figure 3). This finding suggests that the proliferative response elicited by the optimized hormone concentrations remains within a physiologically tolerable range and is constrained by intrinsic tissue-level regulatory mechanisms that preserve normal gonadal architecture. Consistent with this interpretation, Piprek et al. (2012) demonstrated in five anuran species that sex steroids at concentrations within the effective physiological range promoted germ cell proliferation and directed gonadal differentiation without inducing structural abnormalities, whereas supraphysiological doses resulted in gonadal dysgenesis, disrupted corticomedullary organization, and intersex phenotypes [7]. Similarly, Tamschick et al. (2016) reported that high concentrations of ethinylestradiol caused impaired gonadal development in anuran species, whereas lower concentrations induced feminization without structural disruption [35]. This concentration-dependent dichotomy—physiological enhancement versus pathological disruption—has also been widely documented in *teleost fish*, where supra-optimal estrogen and androgen concentrations trigger germ cell apoptosis, follicular atresia, and gonadal fibrosis through non-specific receptor binding and disruption of endogenous steroidogenic feedback [40,41,46]. The absence of such abnormalities in the E_2_-40 and TP-80 groups of the present study indicates that 40 μg·L^−1^ E_2_ and 80 μg·L^−1^ TP fall within the physiologically effective window for *R. dybowskii*.

### 4.3. Fundamental Disparities in Transcriptional Regulation Between Male and Female Differentiation Pathways

Transcriptomic dynamic analysis disclosed fundamentally different differentiation pathways: progressive multi-wave reprogramming for ovaries and concentrated burst activation for testes. The E_2_-40 group exhibits two transcriptional peaks—at stage 31 (initiation) and stage 40 (folliculogenesis)—reflecting the stepwise progression from germ cell cyst formation to oocyte maturation. Haselman et al. characterized global gene expression during early gonadal differentiation in *Xenopus* (*Silurana*) *tropicalis*, identifying key transcriptional events at the undifferentiated and differentiation stages. Their finding of stage-specific gene expression cascades is consistent with our observation of critical transcriptional nodes at stages 30–31 and 37–40 in *R. dybowskii* [47]. In contrast, the TP-80 group shows a single burst at stage 40, coinciding with seminiferous tubule morphogenesis. The TP-80 group exhibited a “quiescent–burst” pattern, in which the number of early DEGs remained at a low level, followed by a significant burst of 15,829 DEGs at stage 40, completely synchronized with testicular tubular structure formation. This differential transcriptional dynamic pattern is closely associated with the distinct developmental requirements of male and female gonads. Ovarian development requires sustained regulation of oocyte growth, folliculogenesis, and maturation across multiple stages. In contrast, testicular development involves rapid, coordinated formation of seminiferous tubules and initiation of prespermatogenesis within a narrow developmental window, whereas testicular development involves rapid, coordinated formation of seminiferous tubules and initiation of prespermatogenesis within a narrow developmental window. Specifically, the ovary must continuously support the synchronous development of oocytes at multiple developmental stages, thereby demonstrating multi-wave regulation. In contrast, the testis accomplishes tubular structural remodeling within a specific time frame, resulting in a burst activation pattern [47,48]. In *Eleutheronema tetradactylum*, comparative gonadal transcriptome analyses similarly indicated that ovary-enriched genes are predominantly associated with multi-stage regulation of oocyte maturation, while testis-enriched genes are concentrated in gene families related to synchronized spermatogenic bursts [49]. Consistent with these findings, significant gene expression disparities between male and female gonads have also been reported in *R. dybowskii* during the reproductive period [50]. This cross-species consistency implies that the molecular kinetic differences between male and female differentiation pathways may represent a conserved characteristic of vertebrates [47,48,51,52,53,54].

### 4.4. Functional Divergence and Synergistic Mechanisms of Core Regulatory Pathways

Pathway enrichment analysis has mapped the regulatory network of sex differentiation to three core pathways: steroid hormone biosynthesis, Wnt, and PPAR signaling. Among these, the steroid hormone biosynthesis pathway was most highly enriched at stage 31, encompassing key genes such as *CYP19a1* and *3α-hsd* [55,56]. These genes directly respond to exogenous hormone stimulation and initiate the endogenous hormone synthesis cascade. This finding suggests a potential feed-forward amplification model for hormone-induced sex reversal, in which exogenous hormones may not only directly activate downstream target genes but also upregulate endogenous steroidogenic enzyme genes, thereby establishing a sustained differentiation signal beyond the initial exogenous stimulus. While this model is supported by the concurrent enrichment of steroid hormone biosynthesis pathway genes (e.g., *CYP19a1*, *3α-hsd*) and the sustained expression of female-biased genes throughout the differentiation period in the present study, direct evidence for positive feedback regulation—such as time-course measurements of endogenous E_2_ and testosterone concentrations, or functional validation of transcriptional regulation at target gene promoters—remains to be established in *R. dybowskii*. Analogous feed-forward mechanisms have been proposed in fish sex-reversal models [46,57,58]. Kowalewski et al. (2009) verified that PPAR is involved in regulating the expression of steroidogenic acute regulatory protein (StAR) and gonadal steroidogenesis, offering molecular evidence for the role of the PPAR signaling pathway in sex differentiation [58]. Velez et al. (2013) comprehensively reviewed the functions of the PPAR system in the female reproductive tract, encompassing steroidogenesis, oocyte maturation, and other crucial processes [59]. The female-biased enrichment of the Wnt signaling pathway, which is consistent with high *Rspo1* expression, corroborates its conserved function in ovarian differentiation. Tomizuka et al. (2008) demonstrated that *Rspo1*-knockout XX mice display sex reversal and mammary duct abnormalities [60]; Buscara further proved that goat *Rspo1* overexpression can reverse the sex reversal phenotype of *Rspo1*-knockout mice [61]. In *Pelodiscus sinensis*, *Rspo1* knockdown results in partial sex reversal of genetic females to males, establishing it as an essential factor for female sexual differentiation [62]. These cross-species functional investigations offer robust evidence for the crucial role of *Rspo1* in the ovarian differentiation of *R. dybowskii*. Notably, the PPAR signaling pathway was consistently enriched in both male and female pathways, yet with distinct functional focuses: in the ovary, it predominantly participates in lipid metabolism to supply energy for oocyte maturation; in the testis, it offers metabolic support for tubular structure formation. This regulatory pattern of “shared core pathway, functionally specialized branches” reflects an optimized resource utilization strategy during evolution. In *Pelodiscus sinensis*, transcriptomic analysis of E_2_-induced pseudo-female embryonic gonads also revealed significant enrichment of the PPAR signaling pathway [63]. These findings are consistent with transcriptomic studies in other amphibian species. Piprek et al. (2013) identified genes involved in sex determination and development in *Xenopus laevis* through transcriptome analysis [51]. Haselman et al. (2015) characterized global gene expression during early gonadal differentiation in *Xenopus* (*Silurana*) *tropicalis* [47]. Tang et al. (2021) analyzed the gonadal transcriptome of *Hoplobatrachus rugulosus* to identify sex development genes, revealing enrichment patterns similar to those observed in the present study [48]. Biscotti et al. (2020) provided insights into the evolution of sexual gene networks in sarcopterygians through *Cynops orientalis* transcriptomics [52]. The convergence of findings across these amphibian taxa reinforces the conserved nature of the core sex differentiation regulatory modules identified in *R. dybowskii*.

The six candidate genes identified through pathway analysis, along with their expression patterns and functional annotations, formed a comprehensive regulatory circuit. Among the female-biased genes, *3α-hsd* participates in steroid hormone metabolism, catalyzing the conversion of estrogen precursors [64]; its expression is regulated by sex hormones and demonstrates sexual dimorphism [65]. *Adcy3* regulates oocyte maturation via the cAMP signaling pathway; the cAMP concentration is a key determinant in maintaining the equilibrium between follicle activation and dormancy [66]. *Rspo1*, as a Wnt pathway activator [60], exhibits significant upregulation from stage 31 onwards, functioning as an early marker of female differentiation. Among the genes with male-biased expression, *Sox9* is a core transcription factor for testicular development [67,68]. Androgen treatment can notably upregulate the expression of *sox9*, which plays a crucial role in the testes [69]. Its expression increases continuously after stage 35, fully synchronized with testicular structural development. *Ptgs2*, a rate-limiting enzyme for prostaglandin synthesis that is constitutively expressed in the testis [70], affects spermatocyte proliferation by regulating PGE_2_ synthesis and interacting synergistically with androgens to regulate spermatogonial differentiation [44]. *Shbg* maintains free testosterone, providing a stable hormonal microenvironment for male differentiation [71]. Notably, these genes do not act independently but function within a synergistic regulatory network. In *Pelodiscus sinensis*, knockdown of *Rspo1* led to the upregulation of multiple genes in the male pathway and the downregulation of genes in the female pathway [61], indicating complex regulatory interactions among these genes. Kuhl et al. (2024) identified a Y-chromosome-specific non-coding RNA located in the 5′ regulatory region of the *bod1l* gene in the European green toad [72], revealing the structural variation mechanism of the amphibian sex-determination locus and offering novel perspectives for understanding the molecular basis of amphibian sex determination.

Notably, these six candidate genes are interconnected with the canonical sex-determination genes (*Dmrt1*, *Cyp19a1a*, and *Foxl2*) within a broader regulatory hierarchy [53,54]. In vertebrates, *Dmrt1* and *Sox9* function as mutually reinforcing core transcription factors in testicular differentiation, where *Dmrt1* directly upregulates *Sox9* expression to drive Sertoli cell differentiation and seminiferous tubule formation [17,51,68,69]. Conversely, *Foxl2* and *Rspo1* constitute critical components of the female-pathway regulatory module: *Foxl2* transcriptionally activates *Cyp19a1a* (aromatase), sustaining estrogen biosynthesis essential for ovarian maintenance [18,20,62], while *Rspo1* potentiates Wnt/β-catenin signaling, which in turn stabilizes *Foxl2* expression through a positive feedback mechanism [51,60,61,62]. The female-biased gene *3α-hsd* participates in estrogen precursor metabolism—a biochemical step upstream of *Cyp19a1a*-mediated aromatization [64,65]—while *Adcy3* regulates cAMP-dependent oocyte maturation downstream of the FSH receptor signaling cascade [66]. On the male side, *Shbg* modulates free androgen bioavailability, thereby indirectly influencing the activation threshold of the *Dmrt1-Sox9* axis [71], and *Ptgs2*-catalyzed prostaglandin E_2_ synthesis interacts with androgen signaling to coordinate spermatogonial proliferation and differentiation [44,70]. The coordinated expression patterns of these six genes, together with their known functional links to the canonical sex-determination hierarchy, suggest that hormone-induced sex reversal in *R. dybowskii* operates through the rewiring of an evolutionarily conserved gene regulatory network rather than through isolated gene-by-gene effects [53,54]. However, the precise epistatic relationships among these genes remain to be elucidated through gene-specific functional studies.

### 4.5. Scientific Contributions and Cross-Species Reference Value

Unlike previous amphibian sex regulation studies that mainly focused on model species (e.g., *Xenopus*) or only investigated unidirectional sex induction [8,42], this study is the first to establish a highly efficient, reproducible bidirectional sex differentiation system in the economically important amphibian *R. dybowskii*. Its research value is manifested at two levels. First, it provides key data for investigating the molecular mechanism of sex determination in *R. dybowskii*. Prior anuran sex regulation studies remained largely at the level of phenotypic observation of temperature-induced effects [2]. This study deciphers the regulatory network of sex differentiation from a global transcriptomic perspective, offering a complete candidate gene set for subsequent functional validation. Second, it provides crucial evidence for the evolutionary study of sex determination in amphibians. Existing conservation analyses of vertebrate sex differentiation regulatory pathways are largely based on data from fish, reptiles, and mammals. The core regulatory roles of the steroid hormone biosynthesis, Wnt, and PPAR pathways discovered in this study, along with the conserved features of transcriptional kinetics in male and female differentiation, demonstrate that amphibians share core sex regulation modules with other vertebrates [53,54]. In this context, the W-linked gene *DM-W* in *Xenopus laevis* represents one of the best-characterized amphibian sex-determining genes. *DM-W* acts as a dominant female-determining factor by transcriptionally suppressing *DMRT1*, thereby directing gonadal fate toward the female pathway [73]. Although *DM-W* has so far been identified only within the genus *Xenopus*, its mode of action illustrates a recurrent evolutionary theme: vertebrate sex determination often involves the interplay between opposing male- and female-pathway transcription factors [17,53]. The identification of *Rspo1* as a strongly female-biased gene in *R. dybowskii*, together with the enrichment of the Wnt signaling pathway, raises the possibility that an analogous female-determining mechanism—operating through Wnt pathway potentiation rather than direct *DMRT1* suppression—may function in ranid frogs [51,60,62]. Whether the *DM-W*-type mechanism represents a lineage-specific innovation in pipids or a more widely conserved feature of amphibian sex determination awaits clarification through comparative genomic analyses across anuran families [52,72]. By placing the transcriptomic landscape of *R. dybowskii* within this comparative framework, our study not only fills a critical phylogenetic gap but also generates testable hypotheses regarding the evolutionary plasticity and conservation of vertebrate sex-determination cascades. This research system can also be extended to sex-control breeding of other economically important frog species, such as *R. chensinensis* and *Pelophylax nigromaculatus*, holding broad industry reference value [8,74,75].

### 4.6. Research Limitations and Future Directions

This study did not include transcriptomes from naturally developed male and female gonads as controls. Therefore, it cannot directly discern whether the transcriptomic features of hormone-induced sex reversal completely recapitulate those of natural sex differentiation. However, histological examination confirmed that the gonadal morphology of hormone-treated groups was consistent with that of natural individuals (Section 3.2), and the identified sex-biased candidate genes are conserved female/male marker genes across known vertebrates.

Future studies should incorporate transcriptome data from natural male and female individuals to verify whether the regulatory networks discovered herein fully mimic the normal differentiation pathway. Moreover, the present study deciphered regulatory networks only at the transcriptomic level and did not directly validate the functions of the candidate genes. Subsequent *CRISPR*/*Cas9* gene knockout/overexpression approaches should be employed to clarify the functions of core genes such as *Rspo1* and *Sox9* in *R. dybowskii* sex differentiation and to further validate the upstream–downstream relationships within the regulatory pathways [76].

This study did not address epigenetic regulatory mechanisms. Existing studies indicate that DNA methylation and histone modifications play important roles in amphibian sex determination [2,77]. Future research could employ whole-genome bisulfite sequencing and ChIP-seq to dissect the dynamics of epigenetic modifications during hormone-induced sex reversal, thereby revealing the complete regulatory chain of “environmental signal–epigenetic regulation–gene expression.”

Furthermore, whether high hormone concentrations induce gonadal toxicity was not verified in this study [78]. Future experiments involving apoptosis detection and oxidative stress indicator measurements could elucidate the toxic effect pathways of high-concentration hormones, providing theoretical support for further optimization of treatment parameters.

For selective breeding, future work could investigate whether crossing pseudo-males (genetic females, phenotypic males) induced by 80 μg·L^−1^ TP with normal females can produce all-female offspring, thereby establishing an all-female breeding system that eliminates the need for continuous hormone treatment [7]. This would fundamentally resolve the low female proportion in farmed populations and align with the development goals of green aquaculture [74,75].

## 5. Conclusions

Addressing the core bottleneck of insufficient female proportion in artificial *R. dybowskii* culture and the knowledge gap in the molecular mechanisms of bidirectional sex differentiation in amphibians, this study systematically elucidated the molecular regulatory mechanisms underlying bidirectional sex reversal in *R. dybowskii* induced by 17β-estradiol and testosterone propionate using a combination of histological and transcriptomic approaches.

This study provides new experimental evidence for the molecular regulatory mechanisms of bidirectional sex reversal in anuran amphibians, confirms the coexistence of evolutionary conservation and species-specific regulatory features in core vertebrate sex-determination pathways, and offers empirical data from a key amphibian node for elucidating the evolutionary trajectory of vertebrate sex-determination mechanisms. It defines the critical window periods and core target genes for hormone-induced sex reversal in *R. dybowskii*.

## Figures and Tables

**Table 1:** Hormone concentration levels for each experimental treatment (μg·L^−1^).

	CK	E_2_-10	E_2_-20	E_2_-40	E_2_-80	E_2_-160	TP-10	TP-20	TP-40	TP-80	TP-160
17β-Estradiol (μg·L^−1^)	0	10	20	40	80	160	0	0	0	0	0
Testosterone propionate (μg·L^−1^)	0	0	0	0	0	0	10	20	40	80	160

**Table 2:** Target gene primer sequence.

Gene Name	Primer Name	Primer Sequence (5′→3′)	Product Length (bp)	Annealing Temperature (°C)
*GAPDH*	GAPDH-F	GGACAGTGTGAGATACAGCAGA	283	60
*GAPDH*	GAPDH-R	TGTGAACGACCTCGACCTTG		
*Sox9*	SOX9-F	ATGCCAGTCAGGGTCAACGG	159	60
*Sox9*	SOX9-R	TTTCATTTAGAAGCCTCCACAGC		
*Ptgs2*	PTGS2-F	CGGATTGCTGCTGAGTTTA	209	60
*Ptgs2*	PTGS2-R	TCCTGCCTGTTGTTTGGTA		
*Shbg*	SHBG-F	GGACAGCTCGATTAACACCA	195	60
*Shbg*	SHBG-R	CCAGGCCCAGTTTCTCATA		
*3α-hsd*	3α-HSD-F	CAGTTATTGGATTTGGGACG	177	60
*3α-hsd*	3α-HSD-R	CATCTGCGATCTTCTTGTGG		
*Adcy3*	ADCY3-F	GGCGGTATCGAGTGTCTGA	195	60
*Adcy3*	ADCY3-R	CCTTCTTTATGGTGTTGTTGA		
*Rspo1*	Rspo1-F	AAGCATCCCTTTGCCCTAT	192	60
*Rspo1*	Rspo1-R	GGAAGTTACTCGCTGTTGG		

**Table 3:** Summary of Transcriptome Sequencing Data Quality.

Sample Group	Total Clean Reads (M)	Total Clean Bases (Gb)	Q20 (%)	Q30 (%)	GC Content (%)	Mapping Rate (%)
Control group (C)	42.85 ± 2.36	6.43 ± 0.35	98.85 ± 0.06	96.45 ± 0.12	46.72 ± 0.98	98.23 ± 0.34
E_2_-40 group	43.52 ± 2.18	6.53 ± 0.33	98.84 ± 0.07	96.34 ± 0.18	46.74 ± 1.12	98.41 ± 0.28
TP-80 group	44.18 ± 2.54	6.63 ± 0.38	98.85 ± 0.08	96.42 ± 0.15	46.58 ± 1.21	98.35 ± 0.31

Note: Data are presented as means ± standard deviation.

**Table 4:** Information on sex-biased candidate genes in *R. dybowskii*.

Gene Name	Full Gene Name	Chromosomal Location	Sex Bias	Main Functional Description
*3α-hsd*	3-alpha-hydroxysteroid dehydrogenase	Chr5	Female-biased	Steroid hormone metabolism; involved in the conversion of estrogen synthesis precursors
*Adcy3*	adenylate cyclase 3	Chr8	Female-biased	Signal transduction; catalyzes cAMP synthesis; participates in oocyte maturation
*Rspo1*	R-spondin 1	Chr12	Female-biased	Wnt signaling pathway activator; promotes ovarian differentiation and development
*Ptgs2*	prostaglandin-endoperoxide synthase 2	Chr3	Male-biased	Rate-limiting enzyme for prostaglandin synthesis; involved in the regulation of spermatocyte proliferation
*Sox9*	SRY-box transcription factor 9	Chr2	Male-biased	Key transcription factor for testis development; participates in Sertoli cell differentiation
*Shbg*	sex hormone-binding globulin	Chr15	Male-biased	Sex hormone-binding protein; maintains free testosterone homeostasis

## Data Availability

The original contributions presented in this study are included in the article. Further inquiries can be directed to the corresponding author.

## Supplementary Materials

The following supporting information can be downloaded at https://www.mdpi.com/article/10.3390/ani16152444/s1.

## Abbreviations

The following abbreviations are used in this manuscript:

**Abbreviation**	**Definition**
*3α-hsd*	3-alpha-hydroxysteroid dehydrogenase
*Adcy3*	adenylate cyclase 3
ANOVA	analysis of variance
AR	androgen receptor
cAMP	cyclic adenosine monophosphate
cDNA	complementary DNA
ChIP-seq	chromatin immunoprecipitation sequencing
*CRISPR/Cas9*	clustered regularly interspaced short palindromic repeats/CRISPR-associated protein 9
*CYP19a1*	cytochrome P450 family 19 subfamily A member 1 (aromatase)
DEGs	differentially expressed genes
DESeq2	Differential Expression analysis for sequence count data 2 (software)
*Dmrt1*	doublesex and mab-3 related transcription factor 1
E_2_	17β-estradiol
ECM	extracellular matrix
FDR	false discovery rate
FPKM	fragments per kilobase of transcript per million mapped reads
*Foxl2*	forkhead box L2
*GAPDH*	glyceraldehyde-3-phosphate dehydrogenase
H&E	hematoxylin and eosin
KEGG	Kyoto Encyclopedia of Genes and Genomes
OR	Oviductus Ranae
PCA	principal component analysis
PI3K-Akt	phosphatidylinositol 3-kinase–protein kinase B
PGE_2_	prostaglandin E_2_
PPAR	peroxisome proliferator-activated receptor
PRMT6	protein arginine methyltransferase 6
*Ptgs2*	prostaglandin-endoperoxide synthase 2
qRT-PCR	quantitative real-time polymerase chain reaction
RIN	RNA integrity number
RNA-seq	RNA sequencing
RSEM	RNA-Seq by Expectation-Maximization (software)
*Rspo1*	R-spondin 1
SD	standard deviation
*Shbg*	sex hormone binding globulin
*Sox9*	SRY-box transcription factor 9
StAR	steroidogenic acute regulatory protein
T	testosterone
TGF-β	transforming growth factor-beta
TP	testosterone propionate
Wnt	Wingless/Integrated (signaling pathway)
