# Supplementary material for: Molecular Mechanisms of Gonadal Differentiation Induced by 17β-Estradiol and Testosterone Propionate in Rana dybowskii
Source: Animals (Basel). 2026 Aug 6;16(15):2444. doi: 10.3390/ani16152444 (PMC13465733; doi:10.3390/ani16152444)
Supplement: Supplementary file 1 [file animals-16-02444-s001.zip › animals-4354663-supplementary.pdf]

Table S1. Effects of Estradiol and Testosterone Propionate on Body Weight of *Rana dybowskii Tadpoles* (mg)

Treatment	Weight at 15 day	Weight at 30 day	Weight at 45 day
CK	10.85±5.49a	154.68±48.27a	125.59±55.98a
E_2-10	21.71±15.10b	155.86±45.05a	174.19±52.43b
E_2-20	20.56±7.05b	143.19±39.04a	186.80±45.54b
E_2-40	22.53±4.20b	190.50±44.22b	244.43±46.94c
E_2-80	21.12±2.77b	173.40±41.57b	226.21±32.22c
E_2-160	21.02±4.23b	137.05±37.60a	200.47±33.88b
TP-10	11.40±7.54a	138.72±30.79a	198.57±54.05b
TP-20	13.40±7.81a	167.01±39.52a	172.24±39.48b
TP-40	14.15±4.94a	125.72±39.87a	186.17±46.47b
TP-80	18.02±8.36b	143.81±53.70a	202.38±39.01b
TP-160	18.23±10.94b	147.64±50.89a	165.71±40.77b

Note: Data are mean ± SD. Different lowercase letters denote significant differences among treatments within the same stage (Duncan's test, $P < 0.05$).

Table S2. Effects of Estradiol and Testosterone Propionate on Body Length of *Rana dybowskii Tadpoles* (mm)

Treatment	Length at 15 day	Length at 30 day	Length at 45 day
CK	10.13±1.39a	26.65±4.70a	26.92±4.99a
E_2-10	13.89±2.00c	27.15±2.87b	30.37±4.46b
E_2-20	13.51±1.59c	27.20±3.36b	31.90±3.86b
E_2-40	13.04±1.09c	30.49±3.70c	36.24±3.31c
E_2-80	12.95±0.90c	28.28±2.99b	35.05±2.31c
E_2-160	14.02±1.35d	25.67±3.21a	32.05±3.91b
TP-10	9.80±1.94a	25.47±2.36a	31.96±4.26b
TP-20	11.71±1.12b	28.00±2.79b	30.64±3.98b
TP-40	12.04±1.17b	23.99±3.13a	32.00±3.15b
TP-80	10.94±1.84b	24.94±3.31a	32.47±2.01b
TP-160	12.65±2.17c	25.96±3.72a	32.00±2.58b

Note: Data are mean ± SD. Different lowercase letters denote significant differences among treatments within the same stage (Duncan's test, $P < 0.05$).

Table S3. Effects of Estradiol and Testosterone Propionate on Sex Ratio of *Rana dybowskii Tadpoles*

Treatment	Survivors(%)	Female proportion (%)	Male proportion (%)
CK	86.4±0.9	55.83±8.33e	44.17±8.33c
E_2-10	87.6±2.2	75.00±5.77f	25.00±5.77b
E_2-20	87.2±1.8	76.67±6.09f	23.33±6.09b
E_2-40	88.8±1.8	93.33±5.44g	6.67±5.44a
E_2-80	87.6±1.7	75.00±4.30f	25.00±4.30b
E_2-160	88.0±2.0	80.83±8.33f	19.17±8.33b
TP-10	87.6±1.7	47.50±1.67d	52.50±1.67d
TP-20	87.2±1.1	37.50±7.88c	62.50±7.88e
TP-40	86.4±0.9	14.17±5.69b	85.83±5.69f
TP-80	88.4±1.7	0.00±0.00a	100.00±0.00g
TP-160	88.0±2.0	0.00±0.00a	100.00±0.00g

Note: Data are presented as number of individuals and corresponding percentages. Different lowercase letters indicate significant differences among treatments (Duncan's test, $P < 0.05$).
